# Western Bluetongue virus serotype 3 in Sardinia, diagnosis and characterization

**DOI:** 10.1111/tbed.13156

**Published:** 2019-03-19

**Authors:** S. Cappai, S. Rolesu, F. Loi, M. Liciardi, A. Leone, M. Marcacci, L. Teodori, I. Mangone, S. Sghaier, O. Portanti, G. Savini, A. Lorusso

**Affiliations:** ^1^ Istituto Zooprofilattico Sperimentale della Sardegna Cagliari Italy; ^2^ OIE Reference Laboratory for Bluetongue Teramo Italy; ^3^ Istituto Zooprofilattico Sperimentale dell'Abruzzo e del Molise (IZSAM) National Reference Center for Whole Genome Sequencing of microbial Pathogens: Database and Bioinformatic Analysis Teramo Italy; ^4^ Laboratoire de virologie Institut de la Recherche Vétérinaire de Tunisie (IRVT) Univérsité de Tunis El Manar Tunis Tunisia

**Keywords:** Bluetongue virus serotype 3, characterization, diagnosis, next‐generation sequencing, Sardinia

## Abstract

Over the last 20 years, Italy has experienced multiple incursions of different serotypes of Bluetongue virus (BTV), a *Culicoides*‐borne arbovirus, the causative agent of bluetongue (BT), a major disease of ruminants. The majority of these incursions originated from Northern Africa, likely because of wind‐blown dissemination of infected midges. Here, we report the first identification of BTV‐3 in Sardinia, Italy. BTV‐3 circulation was evidenced in sentinel animals located in the province of Sud Sardegna on September 19, 2018. Prototype strain BTV‐3 SAR2018 was isolated on cell culture. BTV‐3 SAR2018 sequence and partial sequences obtained by next‐generation sequencing from nucleic acids purified from the isolate and blood samples, respectively, were demonstrated to be almost identical (99–100% of nucleotide identity) to BTV‐3 TUN2016 identified in Tunisia in 2016 and 2017, a scenario already observed in past incursions of other BTV serotypes originating from Northern Africa.

Bluetongue (BT) is a *Culicoides*‐borne OIE‐listed disease which affects ruminants, primarily sheep, caused by Bluetongue virus (BTV), an RNA virus belonging to the genus *Orbivirus*, in the family *Reoviridae* (Maan et al., 2011). BTV genome is composed by 10 segments of linear double‐stranded (ds) RNA, Seg‐1 to Seg‐10, coding for structural and non‐structural proteins. BT has an important impact on livestock industry, mainly due to direct consequences of the viral infection and to the ban of animal trade from infected areas. The spread of the disease is very sensitive to climate conditions, although many management practices mitigate disease transmission and thereby contribute to well‐contained outbreaks (Cappai et al., 2018; Guis et al., 2012). Up to 2008, only 24 classical serotypes of BTV were officially recognized (Maan et al., 2008). These serotypes exist in a complex network of serological cross‐relationships, varying from partial to no protection between heterologous strains. Moreover, in the last years, novel and generally asymptomatic BTV serotypes, also known as atypical BTV serotypes, have been described in the field. Importantly, field outbreaks caused by atypical strains do not require notification to the OIE; thus, they do not cause any restriction to animal trade. Generally, two major geographic groups of BTVs are described and designated as eastern (e) or western (w) topotypes even within the same serotype. The eastern topotype includes viruses from Australia and the Middle/Far East, the western viruses originating from Africa and the Americas (Maan et al., 2008). Since 1998, Southern Europe has experienced multiple incursions of different strains belonging to different serotypes and topotypes of BTV. Strains of BTV‐1e, BTV‐4w, BTV‐9e and BTV‐16e have all entered the eastern Mediterranean region. In addition, BTV‐1w, BTV‐2w and BTV‐4w have entered southern Europe because of wind‐driven dissemination of infected vectors from Northern African countries. Specifically, the virus had been likely introduced to Europe from Northern Africa via two major gateways: (a) from Morocco to Spain through the Straits of Gibraltar and (b) from Tunisia to Italy through Sicily or Sardinia (Cappai et al., 2018; Loi et al., 2017; Lorusso et al., 2013; Wilson & Mellor, 2008). In this regard, Sicily and Sardinia have always been recognized as “points of entry” of BTV in the Mediterranean basin from Northern African countries (Calistri et al., 2004). In November 2016, a novel BTV‐3w (BTV‐3 TUN2016, GenBank accession numbers KY432369‐KY432378) was identified in a symptomatic sheep located in the Municipality of Hannous (Governorate of Nabeul; Delegation of Beni Khalled), in the north‐eastern part of Tunisia, in the middle area of Cap Bon (Sghaier et al., 2017). Further surveillance conducted in Tunisia revealed the widespread circulation of BTV‐3w in ruminants and the existence of an additional variant named BTV‐3 TUN2016/Zarzis (Lorusso et al., 2018). One year after the first official notification in Tunisia, a BTV‐3w strain identical to BTV‐3 TUN2016 was identified in a 3‐year‐old female crossbred sheep belonging to a flock of 400 animals located in the surroundings of Trapani, western part of Sicily, facing the peninsula of Cap Bon (Lorusso et al., 2017). Following guidelines of the national surveillance plan for BTV, on September 19, 2018, serum samples were collected from nine sentinel animals including goats and sheep located in a farm in the municipality of Teulada, province of Sud Sardegna (Sardinia region, Figure 1, blue triangle). The animals previously tested serologically negative for BTV in April, May, June, July and August of the same year. Serum samples were tested by c‐ELISA (Lorusso et al., 2016) at the local Istituto Zooprofilattico Sperimentale (IZS). One sample was positive for the presence of BT antibodies; therefore, in order to detect viral RNA, EDTA blood samples were collected from all animals and purified nucleic acids were tested by real‐time RT‐qPCR which detects Seg‐10 of all known existing BTV serotypes (qPCR_NS3_, Hofmann, Griot, Chaignat, Perler, & Thür, 2008). A total of 4 out of 10 blood samples tested positive by qPCR_NS3_ (threshold cycles (C_*T*_) range 25–28). Positive samples were sent to the National Reference Centre and OIE Reference Laboratory for Bluetongue of the Istituto Zooprofilattico Sperimentale dell'Abruzzo e Molise (IZSAM) for confirmation by qPCR_NS3_ and genotyping. Genotyping was performed by means of the LSI VetMAX European BTV Typing Kit which detects BTV serotypes (BTV‐1, ‐2, ‐4, ‐6, ‐8, ‐9, ‐11 and ‐16) which have been circulating, in previous years, in Europe and in the Mediterranean basin (qPCR_typing_, Life Technologies, Lissieu, France). However, samples were negative by qPCR_typing_. Therefore, based on the epidemiological scenario previously described (Lorusso et al., 2017, 2018) samples were tested by a real‐time RT‐qPCR specific for detection of the Seg‐2 of BTV‐3w (Lorusso et al., 2018). All four samples including 2018TE20820/1, 2018TE20820/4, 2018TE20821/1 and 2018TR20821/2 were positive for the presence of BTV‐3w RNA and were used for direct next‐generation sequencing (NGS) by metagenomic approach in order to obtain information on viral genome sequence and constellation. EDTA blood samples were also processed for virus isolation. Blood samples were washed (3 × in PBS) and, after a freeze and thaw cycle at −80°, used for virus isolation onto confluent monolayers of KC (*Culicoides sonorensis* cell line, Wechsler, Mc Holland, & Tabachnick, 1989) cells for 10 days at 28°C followed by two passages on confluent monolayers of Vero (African green monkey) cells at 37°C, 5% CO_2_. Total RNA extraction from blood samples was performed as previously described (Savini et al., 2017) with some modifications. After cold lysis of erythrocytes and a centrifugation step at 13,000 *g* for 15 min, 200 μl of the supernatant was recovered for RNA extraction and DNAse digestion by using the column‐based Direct‐zol^™^ RNA MiniPrep Plus kit (Zymo Research, Irvine CA) following manufacturer's guidelines. About 30 ng of RNA, quantified with the Qubit^®^ RNA HS Assay Kit (Thermo Fisher Scientific, Waltham, MA), was used for the assessment of the sequence‐independent, single‐primer amplification (SISPA) method (Allander et al., 2005). The SISPA and NGS protocols using the Illumina NextSeq 500 have been previously described in detail by our group at the Istituto Zooprofilattico Sperimentale dell'Abruzzo e Molise (IZSAM, Marcacci et al., 2016; Savini et al., 2017; Marcacci et al., 2018). RNA purified from the cell culture isolate originating from 2018TE20820/4 was also sequenced by NGS.

**Figure 1:**
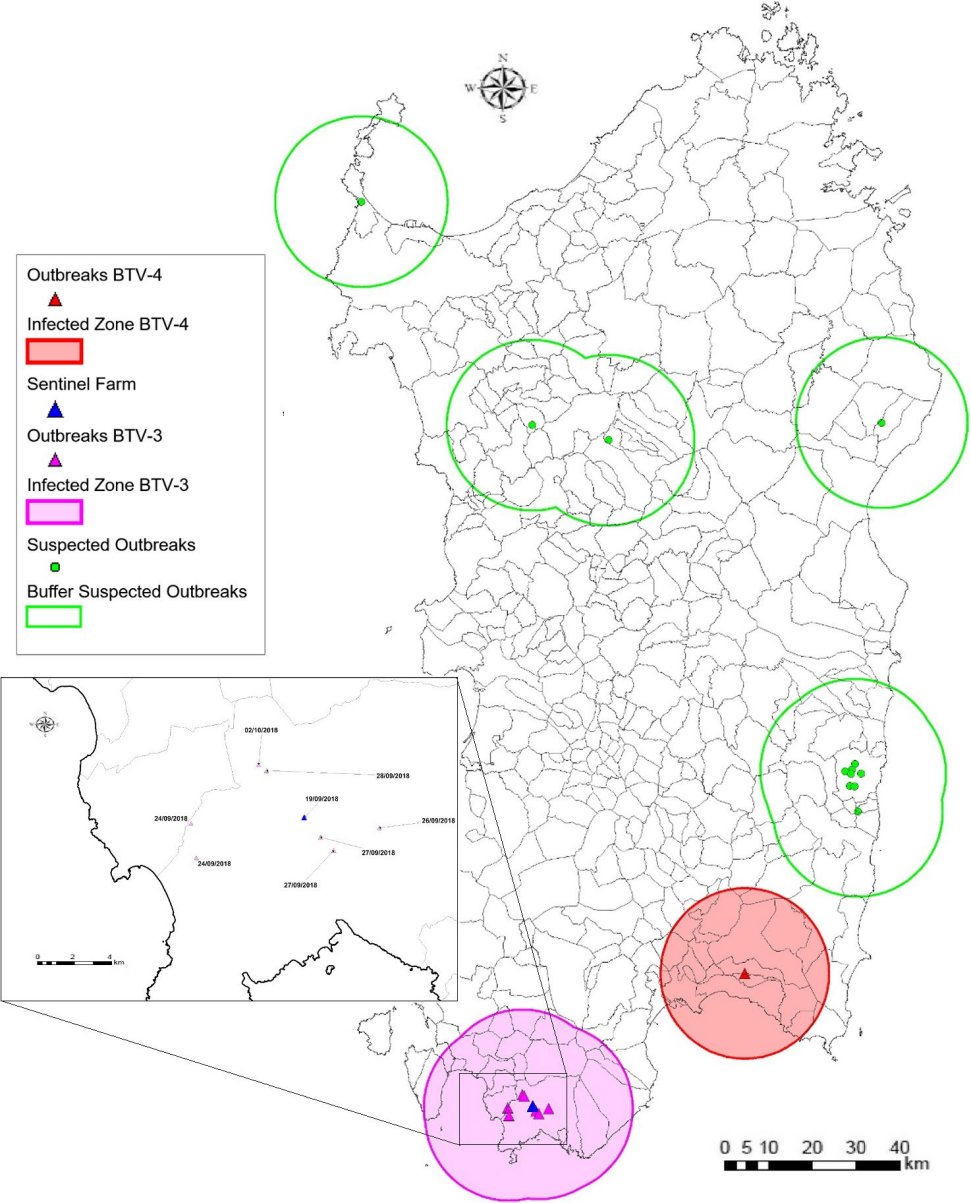
Map illustrating the confirmed or suspected BTV outbreaks (10th October 2018). Inset: location of the farms involved in the BTV‐3w outbreaks in the surroundings of the municipality of Teulada [Colour figure can be viewed at http://www.wileyonlinelibrary.com/]

Between September 24 and 28 2018, the veterinary services of Sardinia region reported clinical signs including depression, fever and nasal discharge (Figure 2) suggesting BTV infection in several animals from four different farms in the same municipality as the sentinel animals. Four sheep died because of the infection. Other animals belonging to the same farms also showed clinical signs. EDTA blood samples were collected and purified nucleic acids tested by qPCR_NS3_. Resulting positive samples were also sent to the IZSAM for confirmation and genotyping by sequencing a small portion of the Seg‐2 of the viral genome using primers previously described (Lorusso et al., 2018). On October 5, 2018, the Italian Ministry of Health notified the World Organization for Animal Health (OIE) of the BTV‐3w outbreak. At the time of writing of this report (October 10 2018), a total number of 8 BTV‐3w outbreaks have been notified in the municipality of Teulada (Figure 1, pink triangles) involving more than 100 small ruminants.

**Figure 2:**
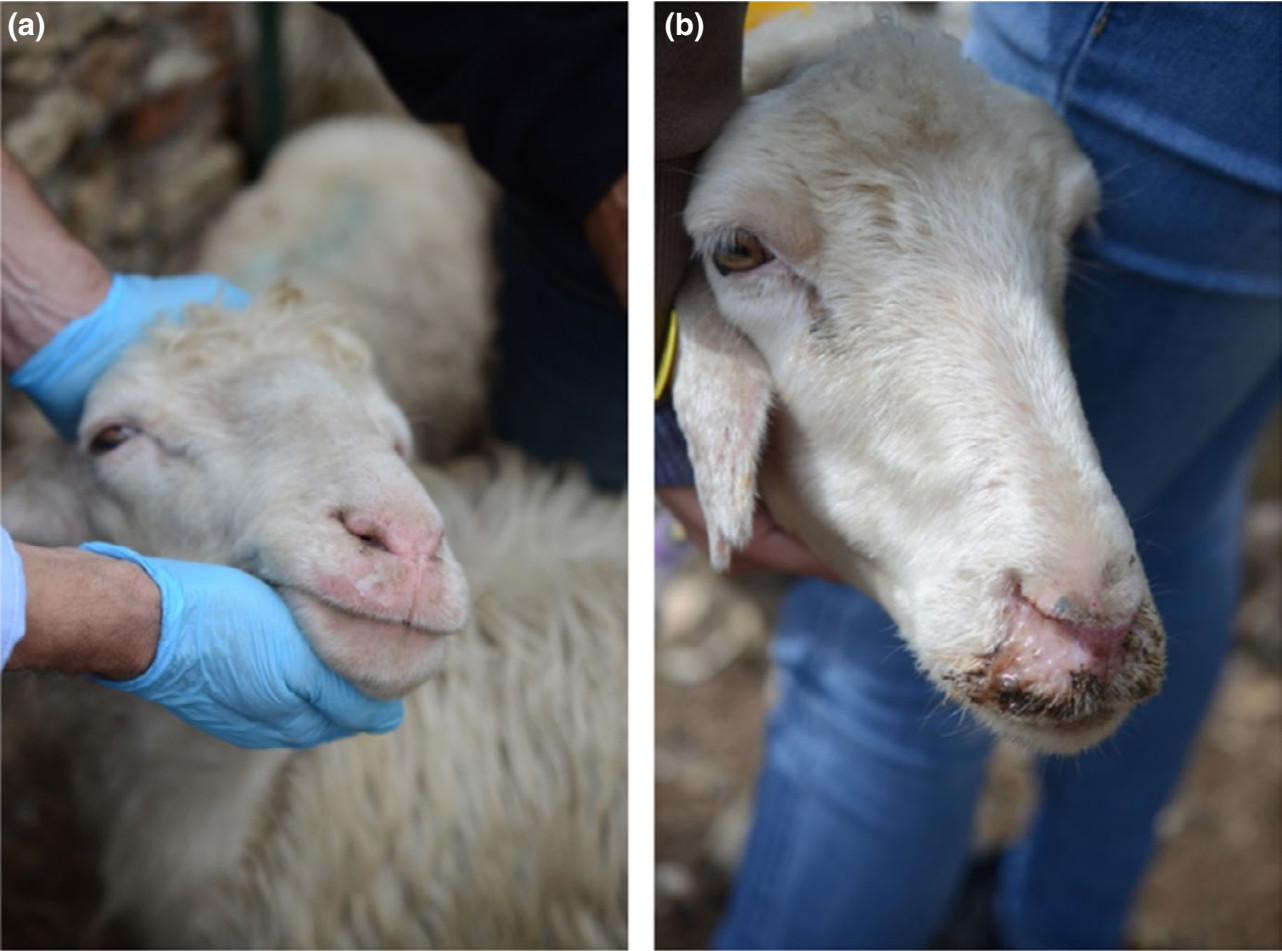
Submandibular oedema, nasal discharge (a) crusted nasal mucosa and discharge (b) observed in two Sarda BTV‐3w‐positive individuals [Colour figure can be viewed at http://www.wileyonlinelibrary.com/]

Sequencing of the four samples (2018TE20820/1, 2018TE20820/4, 2018TE20821/1 and 2018TR20821/2) produced a total number of 2.017.688, 2.198.176, 638.390 and 430.984 paired‐end reads respectively. After quality control and trimming performed using an *in house* script, a total number of 328.774, 744.026, 185.768 and 221.558 reads remained with mean phred scores ranging from 31 to 33. Reads were used for *de novo* assembly by SPAdes v.3.12. The assembly of sample 2018TE20821/2 did not show any contings belonging to BTV. Only partial sequence of the Seg‐4 was detected in sample 20820/1 and 20821/2. On the other hand, the sequencing run of sample 20820/4 produced a total number of 550 contigs longer than 300 nucleotides. Of these, 16 belonged to BTV. This assembly was refined by mapping the reads and contigs against the closest reference genome BTV‐3 TUN2016 (acc. nos. KY432369‐KY432378). The final assembly consisted of partial sequences of all the segments with a mean coverage of 18x.

Cytopathic effect of BTV‐3 SAR2018 was visible at the first cell passage on Vero cells inoculated with the supernatant of KC cells infected with blood sample 2018TE20820/4. As expected, sequence data obtained from the RNA purified from BTV‐3 SAR2018 were of higher quality (data not shown) and importantly contained the complete coding genome information for 10/10 segments of the virus. Obtained sequences were deposited with the GenBank database (accession numbers. MK348537‐MK348546). The analysis was performed on the Phylogeny.fr platform (Dereeper et al., 2008). Seg‐2 sequence of BTV‐3 SAR2018 and western BTV‐3 strains available on GenBank were aligned with MUSCLE (v3.8.31, Edgar, 2004) configured for highest accuracy (MUSCLE with default settings). After alignment, ambiguous regions (i.e. containing gaps and/or poorly aligned) were removed with Gblocks (v0.91b) using the following parameters: minimum length of a block after gap cleaning: 10, no gap positions were allowed in the final alignment, all segments with contiguous non‐conserved positions bigger than 8 were rejected, minimum number of sequences for a flank position, 85% (Castresana, 2000). The phylogenetic tree was reconstructed using the maximum likelihood method implemented in the PhyML program (v3.1/3.0 aLRT; Guindon & Gascuel, 2003; Guindon et al., 2010). The HKY85 substitution model was selected assuming an estimated proportion of invariant sites (of 0.465) and 4 gamma‐distributed rate categories to account for rate heterogeneity across sites. The gamma shape parameter was estimated directly from the data (gamma = 2.751). Reliability for internal branch was assessed using the aLRT test (SH‐Like). Graphical representation and edition of the phylogenetic tree were performed with TreeDyn (v198.3; Chevenet, Brun, Banuls, Jacq, & Chisten, 2006).

BTV‐3 SAR2018 was demonstrated to be almost identical (99% of nucleotide identity in Seg‐1, ‐3, ‐4, ‐5, ‐6, 9 and ‐10; 100% in Seg‐2, ‐7 and ‐8) across all segments to BTV‐3 TUN2016 identified in Tunisia for the first time in November 2016. Likewise, also partial Seg‐2 sequences had 100% of nucleotide identity with that of BTV‐3 TUN2016. The close relationship between BTV‐3 TUN2016 and BTV‐3 SAR2018 is also evident in the Seg‐2 phylogenetic analysis (Figure 3). BTV‐3 SAR2018 clearly clusters with BTV‐3 TUN2016 and it is more distantly related (94.1% of nucleotide identity) to BTV‐3 TUN2016/Zarzis, an additional BTV‐3w strain discovered in 2016 in the eastern part of Tunisia nearby the border with Libya (Lorusso et al., 2018). In turn, BTV‐3 TUN2016/Zarzis is more closely related to a BTV‐3w strain detected in Egypt in 2016 and recent BTV‐3w strains isolated in Israel.

**Figure 3:**
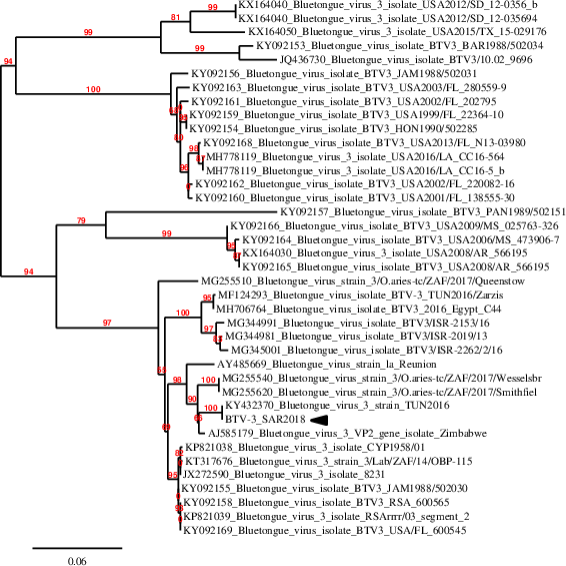
Seg‐2 phylogenetic tree of western strains of BTV‐3. The tree was reconstructed using the maximum likelihood method implemented in the PhyML program (http://www.phylogeny.fr/). Parameters of analysis are available within the text. Eastern BTV‐3 strains have not been included. Bootstrap values ≥70 have been provided at the corresponding nodes. Accession numbers have been also provided. BTV‐3 SAR2018 is indicated by a black arrow [Colour figure can be viewed at http://www.wileyonlinelibrary.com/]

In this report, we have provided evidence for the first incursion of BTV‐3w in Sardinia. The Sardinian strain (BTV‐3 SAR2018) was almost identical to BTV‐3 TUN2016 identified in Tunisia in 2016 and 2017, a scenario already observed in past incursions of other serotypes of BTV originating from Northern Africa. However, BTV‐3 TUN2016 was already detected in one sheep in Sicily in November 2017. Further surveillance activities conducted in Sicily on serum and blood samples of 2197 ruminants (cattle and sheep) living in the areas located within a 20‐km radius around the outbreak farm, demonstrated the absence of BTV‐3 circulation. Only one additional sheep on the outbreak farm tested serologically positive for BTV‐3 (serum neutralization titer, 1:40). Considering that, according to the Italian National Database for Animal Identification and Registration managed by IZSAM (NDB, https://www.vetinfo.it/), no ruminants were moved from Sicily to Sardinia since October 2017, we strongly believe that BTV‐3w emergence in Sardinia was linked to the wind‐borne transportation of infected midges from North Africa. In this regard, further analysis using atmospheric dispersion models are currently being performed in order to establish the most likely period of introduction of infected midges to Sardinia. At first glance, analysis of meteorological data retrieved from an online climatic repository (http://www.forecast.io), taking into consideration the C_*T*_ values, the time at which the virus was first detected in Sardinia, and the winds/dust storms originating from North Africa (available at https://worldview.earthdata.nasa.gov), indicated that the introduction of BTV‐3w to Sardinia likely occurred in the first week of September 2018.

NGS was used to obtain partial sequences directly from blood samples, as well as complete coding sequences from isolated virus. This showed high levels of identity between the BTV TUN2016 and the BTV‐3 SAR2018 strains confirming that NGS is becoming a central and crucial diagnostic technique enabling the identification and characterization of a given BTV strain (and thus its potential origin) in a rapid time period as described in previous reports (Marcacci et al., 2018; Savini et al., 2017). This aspect, combined with the availability of portable third generation sequencers (Beato et al., 2018; Peserico et al., 2019) would make possible the molecular diagnosis of BTV also in field conditions. BTV‐3w circulation was detected in sentinel animals before the onset of the disease in other susceptible animals. Sentinel animals are a key factor of the Italian surveillance programme for BTV, which was implemented nationwide in 2001. According to the sentinel surveillance programme, animals are monthly bled and tested for BTV antibodies, indicating seroconversion. In this regard, it is important to point out that following a recent analysis of risk factors involved in the spread and persistence of BT in Sardinia (Cappai et al., 2018; Loi et al., 2017) the sentinel plan was recently re‐evaluated. Sentinels were re‐located to areas which were described to be at higher risk of novel BTV incursions. This situation likely allowed for the quick detection of the virus by seroconversion before other susceptible animals were infected. Due to the current unavailability of inactivated BTV‐3 vaccine, this virus represents, for the next vector season, a great concern to the livestock industry of Sardinia and Southern Europe (Lorusso et al., 2018). At the time of writing, more suspected outbreaks of BTV have been reported by the Veterinary Services (Figure 1, green dots). In this regard, a more detailed and updated description of BTV‐3w spread in Sardinia is currently in preparation. In conclusion, further efforts are warranted to manufacture a specific inactivated BTV‐3 vaccine and to strengthen collaborations between at‐risk countries.
